# A qualitative study exploring early experiences of misophonia through firsthand perspectives

**DOI:** 10.3389/fpsyt.2026.1852664

**Published:** 2026-08-07

**Authors:** Emily C. Gates, Alyssa Gabbidon, Grace A. Heppes, M. Zachary Rosenthal

**Affiliations:** 1 Department of Psychiatry and Behavioral Sciences, Center for Misophonia and Emotion Regulation, Duke University Medical Center, Durham, NC, United States; 2 Department of Psychology and Neuroscience, Duke University, Durham, NC, United States

**Keywords:** etiology, lived-experience, misophonia, qualitative, reflexive

## Abstract

**Introduction:**

Misophonia is characterized by highly aversive affective, physiological, and behavioral responses to specific auditory and related cues. Although research on misophonia has increased in recent years, relatively little is known about onset, course, and maintenance of this disorder. The present study explores early experiences of misophonia through firsthand perspectives with the goal of constructing themes to reflect patterns related to symptom onset, progression, coping, and perceived causal or contextual factors.

**Methods:**

Adults residing in the United States who identified as having misophonia completed an online survey that included demographic questions, the Duke Misophonia Questionnaire (DMQ), and a series of open-ended questions about early experiences with misophonia. Responses from 50 participants who completed the survey were analyzed using reflexive thematic analysis to generate patterns of meaning across narratives.

**Results:**

A total of seven themes were developed: 1) Concealment, 2) Psychological Distress, 3) Course Variability, 4) Heterogeneity of Misophonic Cues, 5) Coping Mechanisms and Management Strategies, 6) Barriers to Coping, and 7) Theories of Causality.

**Discussion:**

Findings highlight the complex and heterogeneous nature of early experiences and are consistent with a biopsychosocial understanding of misophonia in which contextual and individual factors may shape its development and course.

## Introduction

Misophonia is a newly recognized disorder characterized by highly aversive responses to specific sounds and their associated stimuli (1). These sounds (often referred to as “triggers”) are commonly oral and/or facial (e.g., chewing, throat clearing, gum popping, sniffling, etc.), repetitive, and produced by others (e.g., 2). Trigger sounds can also be non-oral/facial (e.g., pen clicking, typing, and tapping) or environmental sounds not produced directly by people (e.g., clock ticking, mechanical sounds, etc.). Responses to these sounds can vary temporally within or across people and occur before, during, and/or after exposure to triggering cues (e.g., 3). Additionally, variability in responses to these triggers can be influenced by contextual factors, including those that are biological (e.g., accumulated stress, hunger, fatigue, etc.) or situational (e.g., acoustic properties of the physical environment, perceived source of the trigger sound; 4–7).

Characteristic symptoms of misophonia can be organized across biopsychosocial domains using the acronym BASIC (8): Behavioral (e.g., avoidance and/or escape from triggering contexts); Attentional (e.g., hypervigilance toward triggering cues and/or difficulties controlling attention when triggered); Somatic (e.g., hyperarousal such as increased heart rate, muscle tension, sweating or other responses to anticipated or actual triggering cues); Interpersonal (e.g., inhibited communication, hostility and/or indirect aggression with others); Cognitive (e.g., internalizing or externalizing cognitions, rumination, problematic patterns of thinking). In people with clinically elevated misophonia, these BASIC patterns are longstanding (e.g., many months or years), significantly psychologically distressing, and/or cause impairment in important areas of daily functioning (1).

Although prevalence estimates of misophonia vary depending on methodologies, multiple studies from around the world have found that misophonia is not rare. For example, the largest and only population-level representative prevalence study in the U.S. found that 4.6% of adults had clinically elevated symptoms of misophonia (9). Other population level studies done in Germany reported a prevalence rate of clinically elevated misophonia in approximately 2-5% of adults (10, 11). Over the past several years, there has been a rapidly growing body of literature investigating misophonia, but many fundamental questions remain unanswered. To our knowledge, there is little research directly exploring causal factors in etiology, onset, and course of misophonia. As a critical first step to gaining a deeper understanding of its nature and features, significantly more research is needed to understand first-person, subjective accounts as directly experienced by the individuals themselves, known as lived experience, in addition to clinician-observations and objective measurements (12).

In other areas of research, studies have found that incorporating lived experience perspectives into research designs can improve overall quality and produce outcomes that are more beneficial for both patients and clinicians (12, 13). Spencer et al. (14) explored challenges faced in developing interventions for children with misophonia and notably identified the lack of lived experience involvement as a key obstacle. To our knowledge, only a handful of qualitative studies have explored this phenomenon through firsthand perspectives.

One study surveyed the parents of children and adults with misophonia and asked about their perceptions of treatment options available at the time of data collection (15). Most respondents were not professionally diagnosed with misophonia and dissatisfied with the treatments available. Out of those treatments, participants found lifestyle modifications and audiological treatments involving noise cancellation to be the most appropriate. Exposure therapy was considered the least appropriate treatment among both parents and adults with misophonia.

Ozuer et al. (16) conducted two studies to explore meanings affiliated with trigger sounds in those with misophonia. The first study included two focus groups consisting of eight individuals total with misophonia. Using a reflexive thematic analysis, researchers developed eight themes from the focus group transcripts organized into two categories. The first four themes referred to the common meanings associated with trigger sounds: intrusion, violation, offense, and lack of autonomy. The next four themes referred to emotional responses to aversive sounds: anger/defensive rage and disgust referred to immediate emotional responses, while fear and safety behaviors referred to less spontaneous emotions and behaviors associated with misophonia-related situations.

Using a different methodology to explore lived experience perspectives about the daily impairment in functioning, Prabhu et al. (17) asked 51 individuals with misophonia two open-ended questions addressing the problems and effects caused by misophonia. Responses were coded using the World Health Organization’s (WHO) International Classification of Functioning, Disability, and Health (ICF) framework. Results revealed that activity limitation and participation restriction were the most affected domains of functioning among participants.

In another study, Guzick et al. (18) conducted interviews with 20 youth with misophonia and their parents to identify cognitive behavioral processes associated with misophonia and how they impact functioning and family dynamics. Themes included individual internalizing and externalizing processes, secondary emotional and functional consequences, and significant impairment academically, socially, and in family life. Each theme included several subthemes.

From this same dataset, another study thematically analyzing the data from the interviews conducted in Guzick et al. (18) focused on child and parent priorities for advocacy, research, and treatment. Eight themes were constructed: Advancing Awareness for Advocacy, Misophonia is Outside a Child’s Control, Practical and Misophonia-Specific Therapies Needed, Understanding the Neurobiology of Misophonia, Defining Misophonia Beyond Sound Triggers, Understanding the Daily Impact of Misophonia, Uncovering the Misophonia-Mental Health Connection, and Long-Term Prognosis (19).

Additional thematic analysis was performed from this dataset to better understand experiences seeking treatment. Several themes were similar to findings in previously mentioned studies (15, 19) including Minimal Awareness, Few Knowledgeable Professionals, Limited General Resources, No Standard Treatment, Misdiagnosis, and No Official Misophonia Diagnosis. Additionally, themes included Emotional Impact of Treatment-Seeking and two positive experiences, Relaxation and Validation from Providers (20).

Finally, most recently, Woolley et al. (21) analyzed open-ended responses from 60 adults with misophonia using inductive content analysis and the Duke Misophonia Interview (DMI; 22) to enhance the understanding of cognitions both while hearing and after hearing trigger sounds. Similar to themes found in Guzick et al. (18), participants reported the following during exposure to trigger sounds: self-critical/self-devaluing thoughts related to one’s reactions to misophonic sounds, externalizing appraisals of others as rude or inconsiderate, intrusive aggressive thoughts, and escape-focused cognitions. Themes included regret and guilt about their reactions, social worries about how others perceived them, and intrusive or repetitive memories of prior sound experiences.

Collectively, the existing literature examining lived experience has produced valuable insights regarding perceptions of treatment options and experiences seeking them, meanings and cognitions associated with trigger sounds, impact on daily and family functioning, and priorities for advocacy, research, and treatment. However, there is a lack of research focused on early experiences of misophonia, including perceived symptom onset, developmental course, and contextual or moderating factors. Addressing this gap is an important step toward informing theoretical models of etiology and development.

The current study investigates early experiences of misophonia through firsthand perspectives, to better understand etiology, maintenance, and developmental course. Specifically, symptom presentation, progression, and contextual factors were evaluated. The longer-term goal of this study is to yield insights that will contribute to a deeper scientific understanding of misophonia, and the development of evidence-based intervention approaches grounded in lived experience.

## Methods

### Procedure

The study was approved by the Duke Health Institutional Review Board and conducted using an online survey administered by the Duke REDCap data collection platform. Participants were recruited through various Duke Center for Misophonia and Emotion Regulation (CMER) channels including social media accounts, email lists, and the website. Individuals who identified as having misophonia completed the survey, which included a series of questions related to early experiences with misophonia. No protected health information was collected, and participants did not receive compensation. Participation was voluntary, and respondents were free to discontinue the survey at any time.

### Participants

To be eligible, participants had to be at least 18 years of age, reside in the United States, and be able to read and understand English. Participants were provided with the consensus definition of misophonia (1) and were asked whether their experiences aligned. Eligible participants were then given a description of the study and consented to participate electronically. In total, 50 participants completed the study.

### Measures

#### Demographics

Demographic information was collected from all respondents including biological sex, gender identity, age, race, ethnicity, occupation, education, and household income. The sample consisted of 50 participants, with a mean age of 49.2 years (*SD* = 17.4, range = 19–81 years). The mean age of symptom onset was 12.5 years (*SD* = 11.2). The majority were female (80%, *n* = 40) and identified as women (74%, *n* = 37). Of the remaining participants, 20% were male (*n* = 10) and identified as men (*n* = 10), with an additional 6% who identified as agender, non-binary, or genderfluid (*n* = 1 each). The sample was largely homogeneous with respect to race and ethnicity, with 88% identifying as White (*n* = 44) and 96% identifying as non-Hispanic (*n* = 48). Additionally, 66% of participants held at least a college degree (*n* = 33) and the majority reported an annual household income exceeding $100,000 (58%, *n* = 29).

#### Duke misophonia questionnaire

Participants completed the Duke Misophonia Questionnaire (DMQ; 23), a validated self-report measure of misophonia consisting of 86 items across nine subscales assessing trigger frequency, affective, physiological, and cognitive responses, coping strategies, impairment, and beliefs. The DMQ has demonstrated good reliability and convergent and discriminant validity in prior research (23–25). In the present sample, internal consistency was excellent (α = 0.96). The impairment subscale and symptom severity composite score are used quantitatively (23). The mean DMQ Impairment score for the sample was 19.0 (*SD* = 9.4), which is in the clinically elevated impairment range. The mean Symptom Severity Composite score (sum of the affective, physiological, and cognitive responses subscales) was 59.6 (*SD* = 16.3), higher than the threshold for clinically elevated misophonia symptoms on the Symptom Severity Composite (i.e., > 41). Given the variability in severity scores (*SD* = 16.3; 6 participants scored below threshold), the heterogeneity of symptom presentation was considered throughout interpretation, and theme construction drew on the full range of participant experiences.

#### Open-ended questions

Participants were asked to respond to a series of open-ended questions which included the following prompts: 1) What was your earliest memory of misophonia? 2) When you first experienced misophonia, were your primary triggers coming from other people? If so, how would you describe the nature of your relationship(s) with those who triggered you at the time? 3) Have your trigger(s) changed over time? If so, how have your trigger(s) changed over time? 4) Tell us more about what it was like for you when you first began to experience symptoms of misophonia? 5) What was your home environment like at the time you first began experiencing symptoms of misophonia and what ways do you think it may or may not have impacted your experience with misophonia? 6) How did family members and/or those living in your household respond to your experience with misophonia when it first began? 7) Outside of your home, how did other people respond to your experience with misophonia? 8) What do you think caused you to experience misophonia? 9) Once your misophonia symptoms began, what do you think contributed to their continuation? 10) Once your misophonia symptoms began, what do you think contributed to their improvement? Participants were able to provide as much or as little detail as they desired.

### Data analysis

#### Descriptives

Descriptive statistics were used to characterize the demographic and clinical features of the sample. Frequencies and percentages were calculated for categorical variables, while means and standard deviations were calculated for continuous variables, including DMQ subscale and composite scores.

#### Reflexive thematic analysis

Responses to open-ended survey items were analyzed using reflexive thematic analysis (TA), a structured yet flexible method designed to explore patterns of meaning within qualitative data (26, 27). Reflexive TA emphasizes the active role of researchers in data interpretation. Themes are not regarded as inherent within the data, but rather constructed through the researcher’s theoretical perspective, analytic tools, and engagement with the data (28, 29). This method was selected for its appropriateness in exploring subjective, lived experience data. The analysis followed Braun and Clarke’s six-phase framework: 1) data familiarization, 2) code generation, 3) theme construction, 4) theme review, 5) theme definition and naming, and 6) report writing (30). Unlike other forms of thematic analysis, reflexive TA conceptualizes theme generation as an evolving process shaped by repeated interaction with the data (30, 31). An inductive, or “bottom-up,” approach was adopted, meaning the analysis was grounded in the data itself rather than pre-existing theories or hypotheses (26, 30).

Reflexive TA can be conducted from either an experiential or critical standpoint. An experiential approach was chosen, which aims to preserve and reflect the participants’ descriptions of their own experiences (32, 33). The two study team members who conducted the primary analysis (ECG and AG) are research coordinators with direct experience supporting clinical research at the Duke Center for Misophonia and Emotion Regulation. ECG has multiple years of experience leading misophonia research studies and AG has experience supporting clinical research and conducting qualitative analysis using interview and survey data. Both team members were supervised throughout the analytic process by the principal investigator (MZR), a doctoral-level clinical psychologist with extensive experience working with misophonia and a broad range of mental health conditions. Prior to the analysis, study team members began by engaging in reflexive exercises to surface and interrogate assumptions that may have been shaped by their familiarity with the clinical population. With methodological assumptions established, data familiarization and subsequent phases were initiated. The first step involved thoroughly reading through each response and noting key insights. This was performed independently by two study team members. Following familiarization, the purpose of the next phase was to identify salient keywords and phrases within each response. This process led to the construction of potential themes. Researchers then revisited each response to discuss the coherence of its fit within the candidate theme and to engage in reflexive dialogue about how each researcher’s interpretive lens may have shaped the thematic framework. The analysis involved multiple rounds of review and engagement with the reflexive process before finalizing the thematic framework.

Across the full dataset (489 responses from 50 participants), 68 responses were reviewed and excluded from the final framework. Excluded responses fell into one of three categories: 1) brief neutral or positive descriptors of relationships or home environments (e.g., “good relationship,” “stable and supportive”), 2) responses reflecting uncertainty or cross-references to prior answers (e.g., “I don’t know,” “same as above”), and 3) generic restatements of experiences already captured within existing themes. The exclusion of brief positive or neutral descriptors was not intended to presuppose that positive experiences are unimportant in shaping misophonia. Rather, consistent with Braun and Clarke (26, 29), who state that themes should capture shared meaning rather than shared topic, these responses did not yield sufficient depth or patterned meaning to constitute a theme. Notably, positive experiences were represented within the final thematic framework. All 50 participants were represented in multiple themes/subthemes.

## Results

### Development of themes

Reflexive thematic analysis was used to derive seven themes and twenty-two subthemes: 1) Concealment, 2) Psychological Distress, 3) Course Variability, 4) Heterogeneity of Misophonic Cues, 5) Coping Mechanisms and Management Strategies, 6) Barriers to Coping, and 7) Theories of Causality. Themes and subthemes reflect patterns within participants’ narratives of their early experiences with misophonia. **Table 1** provides frequency counts for each theme and subtheme for descriptive context only. Consistent with Braun and Clarke (26, 29), who advise against the over-quantification of thematic frameworks, these counts are offered as a transparency measure to convey the breadth of participant representation across themes and should not be interpreted as indicators of thematic importance, prevalence in the broader population, or ranking among themes.

**Table 1:** Theme and subthemes.

Themes	Unique participants	Number of references
Concealment		
Limited Disclosure	16	21
Suppressing Emotional Responses	28	32
Psychological Distress		
Distorted Self-Perception and Shame	15	18
Heightened Emotional Reactivity	42	52
Co-occurring Mental Health Conditions	7	8
Course Variability		
Symptom Intensification Over Time	46	66
Conditional Relief or Temporary Improvement	9	9
Heterogeneity of Misophonic Cues		
Oral-Specific Triggers	41	49
General Sound and Ambient Noise Triggers	27	31
Triggers Linked to Escape / Entrapment	8	10
Triggers Linked to Family or Domestic Environments	28	35
Coping Mechanisms and Management Strategies		
Avoidance	27	30
Sound Masking	14	15
Recognition and Awareness	9	10
Variability in Intervention Effectiveness	5	5
Barriers to Coping		
Lack of Coping Tools or Strategies	6	6
Limited Information / Awareness	11	14
Absence of Social or Clinical Support	32	45
Theories of Causality		
Uncertainty / Unknown Origins	22	23
Genetic Theories	7	7
Neurological / Sensory Hypersensitivity	15	17
Stressful Childhood or Early Life Experiences	14	20

Participants = number of unique participants with at least one response coded to that theme or subtheme. References = total number of coded responses within a theme or subtheme. Participants could be coded to multiple subthemes within and across themes. Frequency counts are provided for context and should not be interpreted as indicators of thematic importance.

### Theme 1: concealment

The first theme generated from the responses reflects participants’ decisions to conceal their misophonia symptoms, reactions, or diagnosis from others. The subthemes within this theme include 1) Limited disclosure and 2) Suppressing emotional responses.

#### Subtheme 1a: limited disclosure

This subtheme reveals some participants’ decisions to hide their diagnosis from others or to share it only with close family members. Many indicated that people in their lives were unaware of their symptoms. One participant stated, “I hid it very well. Most people didn’t know I was suffering.” Another noted that “no one knew” about their diagnosis and that they “never told a soul” that they had misophonia. Among those who did disclose, sharing was typically restricted to the household. One participant noted, “I have kept it quite discreet outside of my home and don’t really tell anyone else or let anyone know I experience misophonia besides the people in my home.” Another stated, “I have never told anyone outside my home” about their misophonia.

#### Subtheme 1b: suppressing emotional responses

Some participants described hiding the emotional responses caused by misophonia. One participant said, “It was stressful to keep these feelings inside.” Another shared, “I hid it from them but internally I was freaking out.”

### Theme 2: psychological distress

Participants referenced psychological distress associated with their early experiences of misophonia. The subthemes include 1) Distorted self-perception and shame, 2) Heightened emotional reactivity, and 3) Co-occurring mental health conditions.

#### Subtheme 2a: distorted self-perception and shame

This subtheme reflects on negative self-perceptions associated with misophonia. One participant described the “self-esteem and worthiness issue” that this disorder has caused them. Another shared how having misophonia made them feel abnormal: “When I was young, I just assumed something was wrong with me and/or that I was being irrational. It didn’t make sense to me that those sounds did not bother everyone else.” Another participant stated, “Nobody has ever heard of it; psychologists, therapists, psychiatrists, and other doctors. I am ashamed and embarrassed.”

#### Subtheme 2b: heightened emotional reactivity

Participants endorsed intense emotional reactions to triggering sounds. One participant reported “pent up feelings and anger.” Another stated, “I can go to have a perfect friendship to hating the person making the sound to be my most hated enemy in a split second.”

#### Subtheme 2c: co-occurring mental health conditions

Some participants mentioned co-occurring mental health conditions. One participant shared, “I have ADHD and I think the sensory aspects that go along with it, along with the hyper focus aspect of it, make not hearing and moving on from uncomfortable noises highly difficult if not impossible.” Another participant noted, “I have been diagnosed with generalized anxiety and depression.”

### Theme 3: course variability

This theme reflects participants’ accounts of changes in misophonia symptoms over time. The subthemes include: 1) Symptom intensification over time and 2) Conditional relief or temporary improvements.

#### Subtheme 3a: symptom intensification over time

Many participants noted that they experienced no improvement or that their symptoms worsened over time. One participant stated that their triggers grew in number: “I think I started recognizing even more sounds after the first time. It started making me more attuned and sensitive to all sounds around me. It felt like after the first time, I was noticing every sound people were making in the house.” Another participant reported that their “triggers diversified,” resulting in heightened anxiety and fixation on situations in which triggers may occur. Participants also described psychosocial factors, including stress and changes in mental health, as contributing to symptom worsening.

#### Subtheme 3b: conditional relief or temporary improvement

Some participants referenced conditional relief or temporary improvement. One participant stated, “I would say my symptoms haven’t necessarily improved, but I have learned to deal with them better. A big help for me now is now that I’m older, I don’t live with any family members.” Another reported that their reactions “seems to be less intense” when their baseline mental health was more stable.

### Theme 4: heterogeneity of misophonic cues

This theme captures the diversity of stimuli identified as triggering misophonic responses. The subthemes include: 1) Oral-specific triggers, 2) General sound and ambient noise triggers, 3) Triggers linked to escape or feelings of entrapment, and 4) Triggers linked to family members or domestic environments.

#### Subtheme 4a: oral-specific triggers

Cues related to mouth and oral sounds were commonly mentioned. One participant said, “Oral sounds really triggered me, and I started to notice them all around.” Another participant shared how even the thought of oral noises triggered a reaction: “My kids know that I don’t want them chewing gum in my presence and it’s become a ridiculously big deal. I don’t even want to see a sliver of mint green in their mouths. It’s as though the very possibility of gum-chewing is enough.”

#### Subtheme 4b: general sound and ambient noise triggers

Participants also referred to being triggered by general sounds or loud noises in their environment. One participant explained how “living in a loud city where there’s overall more noise” made their misophonia worse. Another participant stated that the noises around them “never seemed to stop,” while another reported that they “felt bothered by loud noises”. Other triggering cues included “crinkly noise,” “tapping,” “knuckle cracking,” “sniffing,” and “the sound of Velcro.”

#### Subtheme 4c: triggers linked to escape or feelings of entrapment

Some participants referenced stronger reactions when they felt unable to leave environments with triggering sounds. One participant stated, “The feeling of helplessness. Being attacked while I’m in my own safe space with nowhere to run or hide.” Another participant explained that “not being able to escape the noises” around them heightened their reaction to triggers.

#### Subtheme 4d: triggers linked to family members or domestic environments

Many participants reported that sounds produced by family members are especially triggering. One participant stated, “Being around my younger sister persisted the problem.” Another participant shared, “As for my father, I also love him dearly; he was my primary caretaker when I was a preschooler … but when I was around 12 or so, I could barely stand to be in the same room with him when he ate.” One participant referred to their relationship with their mother, who frequently triggered them, as “very strained,” noting that their mother labeled them as “dramatic.”

### Theme 5: coping mechanisms and management strategies

Participants shared several coping mechanisms and management strategies used to reduce misophonia symptoms. The subthemes include: 1) Avoidance, 2) Sound masking, 3) Recognition and awareness, and 4) Variability in intervention strategies.

#### Subtheme 5a: avoidance

Environmental and social avoidance were commonly referenced. One participant stated that they managed their symptoms by “distancing myself from situations where I knew I was likely to experience symptoms of misophonia.” Another participant noted that “avoiding situations” that might contain triggering sounds helped reduce distress.

#### Subtheme 5b: sound masking

Participants also endorsed the use of sound-blocking or masking strategies. One participant stated, “I try to manage symptoms with noise-cancelling headphones, putting on loud music to drown out triggers.” Another participant reported that “carrying earplugs around” helped them “block out any bad sounds” when around others.

#### Subtheme 5c: recognition and awareness

Some participants felt relief after learning about misophonia or receiving a label for their experiences. One participant stated that after learning about misophonia, it felt like “a light bulb went on.” Another participant noted that “putting a name” to their reactions made them feel “more tolerant and not so crazy.”

#### Subtheme 5d: variability in intervention strategies

Participants reported a range of intervention strategies they had tried or were currently using to manage their misophonia. One participant stated, “As I have gotten older, I have experience with CBT, DBT, and mindfulness that have been helpful at times.” Another participant noted that religion helped them cope: “I also have grown a relationship with Christ which has helped me see the way my misophonia has grown in me.” Participants also described strategies that had not been effective. One participant noted that “all masking/soundproofing attempts turned out futile.” The diversity of strategies described, ranging from formal psychotherapeutic approaches to spiritual and personal practices, reflects the individualized and often self-directed nature of coping with misophonia in the absence of established, evidence-based treatments.

### Theme 6: barriers to coping

Participants also noted factors that made coping with misophonia more difficult. The subthemes include: 1) Lack of coping tools or strategies, 2) Limited information or awareness about misophonia, and 3) Absence of social or clinical support.

#### Subtheme 6a: lack of coping tools or strategies

Some participants wrote about having few coping tools or strategies. One noted that having “a lack of behavioral coping skills” contributed to worsening reactions. Another participant reported having “almost no coping or affirmative communication skills.”

#### Subtheme 6b: limited information and awareness of misophonia

Participants also identified limited knowledge about misophonia as problematic. One participant stated that the “lack of knowing how to help” their misophonia made it more difficult to manage. Another participant shared, “I didn’t learn until I was a young adult that other people experience this too … I felt lost as to how to help myself.”

#### Subtheme 6c: absence of social or clinical support

Some participants referenced a lack of social or clinical support. One participant noted that a “lack of support from the people closest to [them]” made management more difficult. Another participant reported that having “no therapy” was challenging.

### Theme 7: theories of causality

Participants shared different beliefs about the possible causes of their misophonia. The subthemes include: 1) Uncertainty and unknown origins, 2) Genetic theories, 3) Neurological and sensory hypersensitivity theories, and 4) Stressful childhood or early life experiences.

#### Subtheme 7a: uncertainty and unknown origins

Many participants expressed uncertainty about the cause of their misophonia. One participant stated they had “no idea as to the origin of this behavior.” Another participant noted, “I can’t think of a specific event or trauma that would spark misophonia, it almost feels like I was born with it.”

#### Subtheme 7b: genetic theories

Some participants referenced genetic explanations. One participant stated, “Genetics. My biological father was extremely triggered by sound.” Others referenced family members with similar sensitivities to sound.

#### Subtheme 7c: neurological and sensory hypersensitivity theories

Participants also mentioned potential neurological or sensory explanations. One participant stated, “I just assume there is something wrong with my brain chemistry that causes this, since it’s a [non]-issue for regular people.” Another participant stated, “I really have no idea, other than possibly having sensitive ears.”

#### Subtheme 7d: stressful childhood or early life experiences

Some participants described stressful childhood environments as possible contributing factors. One participant stated, “I do believe my home environment had an impact. My family was under immense stress.” Another described growing up in “a high stress environment” marked by “parent’s alcoholism and emotional abuse.”

## Discussion

This study explored early experiences of misophonia using reflexive thematic analysis to construct themes related to onset, symptom progression, and contextual factors that may have been influential. This method was chosen due to its appropriateness in reflecting the nature of qualitative data and recognizing the role researchers play in interpreting participants’ responses. Following analysis, seven themes were generated: 1) Concealment, 2) Psychological Distress, 3) Course Variability, 4) Heterogeneity of Misophonic Cues, 5) Coping Mechanisms and Management Strategies, 6) Barriers to Coping, and 7) Theories of Causality. Together, these themes provide insight into how individuals experience, understand, and manage their misophonia.

### Theme 1: concealment

Many participants described deliberately concealing symptoms or limiting disclosure of symptoms to close family, often due to fears of stigma or being misunderstood. A recent mixed methods study also revealed that participants often mask their reactions or hide their condition to avoid judgment (16). This aligns with many of our participants’ responses, reinforcing that social acceptance and the fear of rejection are meaningful factors in early experiences of misophonia.

In many cases, concealment appeared to be closely tied to feelings of invalidation or misunderstanding by others. Participants frequently reported suppressing their reactions in social settings or withholding disclosure due to concerns about appearing irrational or overly sensitive. These experiences highlight how individuals may internalize negative perceptions of their symptoms, which can contribute to shame, distorted self-perception, and heightened emotional reactivity. Concealment may therefore function as a protective strategy intended to avoid social conflict or embarrassment, but it may also limit opportunities for support and connection with others.

### Theme 2: psychological distress

The psychological distress associated with misophonia is well documented and continues to emerge as a key feature of this disorder. Our findings are consistent with literature describing elevated levels of anxiety, anger, and emotional dysregulation among individuals with misophonia (34–36). Participants frequently described feelings of shame, abnormality, and confusion about their reactions to certain sounds, particularly during earlier developmental stages when they lacked an understanding of their experiences. Several participants described questioning their own rationality or feeling embarrassed about their reactions, indicating that misophonia may contribute to negative self-appraisals and internalized stigma.

These findings also highlight the variability of biopsychosocial responses associated with misophonia. Participants referenced a range of emotional and behavioral reactions, including anger, irritability, anxiety, and interpersonal strain. The diverse presentation of psychological symptoms (e.g., behavioral, attentional, somatic, interpersonal, and cognitive) suggests that psychotherapy interventions should be flexible and idiographic in nature. Interventions that incorporate evidence-based strategies for underlying transdiagnostic processes, psychoeducation, and validation of lived experience may be particularly beneficial in addressing these symptoms.

### Theme 3: course variability

The overwhelming response to our inquiry about the course of misophonia was that after initial onset, participants consistently reported gradual worsening of symptoms influenced by a range of moderating factors. Many participants described an increase in sensitivity to triggering sounds or an expansion in the number of cues that elicited a response. This perceived progression may reflect increased attentional awareness of auditory stimuli, as individuals become more attuned to specific sounds through repeated exposure and negative emotional associations.

Participant reports suggest that the perceived progression of misophonia over time is variable and may fluctuate in response to changes in psychosocial stressors, though causal inferences cannot be drawn from retrospective accounts. For example, many participants endorsed temporary relief associated with changes in life circumstances, such as moving homes, not living with certain people, changing jobs, or improvements in baseline mental health. These responses highlight the role of environmental and contextual influences in shaping the experience of misophonia. Changes in living environments or interpersonal dynamics may reduce exposure to frequently occurring triggers, which may contribute to periods of symptom relief. The variability described by participants suggests that misophonia may follow a nonlinear course rather than a fixed trajectory. Retrospective self-report data from this sample reflected this variability, with an onset age range of 4 to 66 years, suggesting that while onset is most commonly reported to occur during childhood or adolescence, the developmental window for symptom emergence varied considerably across individuals. Future longitudinal work is needed to clarify whether patterns of progression, including trigger expansion and stress-related exacerbation, differ based on age of onset. Understanding these patterns of change may decrease hopelessness or worries about the perceived inexorability of lifetime symptom progression.

### Theme 4: heterogeneity of misophonic cues

Reports of aversive, triggering stimuli and their sources varied, but oral-facial sounds produced by family members were especially salient. This aligns directly with a growing body of literature consistently finding that oral and facial sounds produced by others are among the most common triggers. In addition, it also converges with those indicating that sounds produced by family members and close people are among the most distressing (23, 34, 37).

In our study, participants emphasized their experiences with triggers produced by family members and the escalation in symptoms they experience when feeling entrapped or unable to escape the environment. These responses further illustrate how the physical and social contexts in which sounds occur may influence responses. These findings also highlight how interpersonal processes and contextual factors may shape the experience of misophonia. Family environments, shared living spaces, and repeated exposure to specific sound patterns were frequently described by participants as perceived contributors to the development or reinforcement of negative associations with sounds, though the retrospective and self-report nature of these accounts prevents causal interpretation. As such, understanding the relational and environmental context in which triggers occur may provide valuable insight into mechanisms underlying misophonia and inform more comprehensive treatment approaches.

### Theme 5: coping mechanisms and management strategies

Coping strategies included avoidance of triggering environments, use of sound-blocking tools, enhanced self-awareness, and engagement in therapeutic or spiritual resources. These findings are consistent with recent research showing that avoidance is a commonly endorsed strategy (38).

Avoidance may provide short-term relief from distress by reducing exposure to triggering stimuli. However, it may also lead to unintended social consequences, such as limiting participation in shared meals, social gatherings, or work environments. Participants in this study frequently described modifying their routines or environments to reduce exposure to triggers. These responses highlight the ways in which misophonia can shape daily decision-making and engagement in everyday life.

Several participants described the experience of learning about misophonia as a moment of clarity that helped them contextualize their reactions. This sense of recognition appeared to carry meaningful weight, often accompanied by relief and reduction in self-blame. These findings align with qualitative reports suggesting that psychoeducation can reduce self-stigma and provide individuals with a framework for understanding their experiences (16). They also underscore the potential clinical value of early and accessible misophonia awareness and education.

### Theme 6: barriers to coping

Complimentary to our finding that awareness may improve one’s ability to cope, lack of awareness was commonly endorsed as a barrier to coping. This includes not only the individuals’ lack of awareness but also limited recognition among healthcare professionals and within social circles. Participants frequently described experiences of confusion or isolation prior to learning about misophonia, which delayed help-seeking and limited access to appropriate support. This suggests that dissemination of research findings, increased clinical education, and public awareness efforts may be important to improving outcomes for individuals with misophonia. It is also worth noting that the present sample was predominantly highly educated, with 66% of participants holding at least a college degree. The barriers to awareness, help-seeking, and appropriate clinical support endorsed despite their high level of education suggest that these obstacles are likely more pronounced for individuals with less education or access to resources. This underscores the critical importance of broad, accessible public education efforts and the dissemination of research findings beyond academic and clinical settings.

### Theme 7: perspectives on causality

Participants expressed a range of beliefs about the origins of this disorder, including genetic predisposition, neurological or sensory hypersensitivity, and stressful early life experiences. Despite various speculations about possible causes, the most prominent explanation provided in response to this prompt was uncertainty. This pattern of results indicates that there may be no single, specific cause that can be easily identified for all people with misophonia. Instead, it may be expected that, across people with misophonia, there may be a wide range of speculative sources of casualty. This may help explain why, clinically, it is common for patients with misophonia to raise questions about this topic. Participants’ reflections illustrate how individuals attempt to make sense of complex experiences by drawing on personal observations, family histories, or available information. Understanding how individuals conceptualize the origins of their misophonia may also have implications for clinical care. Personal beliefs about causality can shape expectations for treatment, coping strategies, and engagement with mental health services. Future research may benefit from further exploring how these beliefs influence treatment outcomes.

### Limitations and future directions

This study has several key limitations. The sample was predominantly White, female, and highly educated, which significantly limits the generalizability of results. This could be due to the recruitment methods used for this study, which heavily relied on self-selection. People familiar with the term misophonia, the Duke Center for Misophonia and Emotion Regulation, and who sought out information or services for misophonia were more likely to participate. The study was exclusively distributed electronically, so those with greater access to internet connection were more likely to participate as well. It is also imperative to recognize the limitations of a smaller sample size. Although more common in qualitative research, we are beginning to understand the heterogeneity of misophonia presentation and lived experiences, so larger, more representative samples are crucial to understanding the true scope of this disorder.

The operationalization of misophonia and the clinical characteristics of the sample relied on honesty and the subjective interpretation of items rather than a more formal clinical assessment. The self-report nature of the study also introduces potential recall and sequence biases. The use of reflexive thematic analysis provides rich, contextually grounded insights but is inherently interpretive as themes are constructed by researchers. Similar limitations are acknowledged in the qualitative misophonia literature (16).

Results from the present study help pave the way for future studies using mixed methods designs that integrate qualitative and quantitative data, longitudinal designs to more directly capture change over time, and the inclusion of individuals with lived experience throughout project development to circumvent these limitations and advance scientific knowledge and clinical effectiveness for treating misophonia.

### Implications

This study emphasizes the importance of centering lived experience in misophonia research. Insights from participants are consistent with a biopsychosocial framework and highlight contextual and individual factors that participants perceived as influencing their misophonia. The findings of this study suggest that interventions for this disorder should be accessible, scalable, flexible, person-centered, and idiographic in nature as there is variability within people who experience misophonia and lack of resources is a common treatment barrier. They also suggest that recognition and validation of misophonia experiences may influence some of the distress and impairment caused by misophonia. Additionally, social and familial relationships play a key role in the experience of misophonia. Utilizing and building off these insights will enhance theoretical models and ultimately lead to more effective interventions informed by the real-world experiences of the misophonia community.

## Data Availability

The raw data supporting the conclusions of this article will be made available by the authors, without undue reservation.
